# Navigating the Complex Landscape of Fibrodysplasia Ossificans Progressiva: From Current Paradigms to Therapeutic Frontiers

**DOI:** 10.3390/genes14122162

**Published:** 2023-11-30

**Authors:** Saeed Anwar, Toshifumi Yokota

**Affiliations:** Department of Medical Genetics, Faculty of Medicine and Dentistry, University of Alberta, Edmonton, AB T6G 2H7, Canada; sanwar@ualberta.ca

**Keywords:** fibrodysplasia ossificans progressiva (FOP), ultra-rare disorders, heterotopic ossification, bone morphogenetic proteins (BMPs), ACVR1, targeted therapy, genetic therapy, antisense therapy, clinical trial design for ultra-rare diseases

## Abstract

Fibrodysplasia ossificans progressiva (FOP) is an enigmatic, ultra-rare genetic disorder characterized by progressive heterotopic ossification, wherein soft connective tissues undergo pathological transformation into bone structures. This incapacitating process severely limits patient mobility and poses formidable challenges for therapeutic intervention. Predominantly caused by missense mutations in the *ACVR1* gene, this disorder has hitherto defied comprehensive mechanistic understanding and effective treatment paradigms. This write-up offers a comprehensive overview of the contemporary understanding of FOP’s complex pathobiology, underscored by advances in molecular genetics and proteomic studies. We delve into targeted therapy, spanning genetic therapeutics, enzymatic and transcriptional modulation, stem cell therapies, and innovative immunotherapies. We also highlight the intricate complexities surrounding clinical trial design for ultra-rare disorders like FOP, addressing fundamental statistical limitations, ethical conundrums, and methodological advancements essential for the success of interventional studies. We advocate for the adoption of a multi-disciplinary approach that converges bench-to-bedside research, clinical expertise, and ethical considerations to tackle the challenges of ultra-rare diseases like FOP and comparable ultra-rare diseases. In essence, this manuscript serves a dual purpose: as a definitive scientific resource for ongoing and future FOP research and a call to action for innovative solutions to address methodological and ethical challenges that impede progress in the broader field of medical research into ultra-rare conditions.

## 1. Introduction

Fibrodysplasia ossificans progressiva (FOP, OMIM #135100), also known colloquially as stone man syndrome or wood man syndrome, is a debilitating genetic disorder characterized by the progression of heterotopic ossification (HO) that transforms soft connective tissues, e.g., muscles, tendons, and ligaments, into bone through a process known as endochondral ossification [1,2,3]. FOP is considered an ultra-rare disease; its global prevalence varies, ranging from 0.036 per million in the Asia–Pacific region to 0.65 per million in North America [4,5]. However, some European regions, e.g., Sweden and France, report even higher rates of 1.43 and 1.36 per million, respectively [4,5,6]. As the name suggests, FOP encapsulates fibrous dysplasia and the inexorable progression of heterotopic ossification (HO). FOP is the first known medical condition where one organ system changes into another, and it is considered to be a paradigmatic model for understanding the dysregulation of cellular fate commitment and tissue homeostasis [2,3]. Hallmark features of FOP are congenital malformation of the big toes and bilateral hallux valgus deformities [2,3].

At its molecular core, FOP is primarily driven by activating mutations in the gene encoding Activin Receptor A Type I (*ACVR1*), also known as *ALK2* [7]. This receptor is a key component of the bone morphogenetic protein (BMP) signaling pathways [8,9,10,11,12]. In individuals with FOP, mutations in *ACVR1* lead to aberrant BMP signaling and increased responsiveness to the ligand Activin A, catalyzing HO [2,7,13,14]. Clinically, FOP manifests in episodic flare-ups that precede localized HO, initially targeting areas near the axial skeleton before spreading outward [15,16,17,18,19,20]. This spatiotemporal pattern suggests that other unidentified regulatory factors contribute to localized susceptibility to HO. Complications of FOP are not limited to skeletal issues but extend to difficulties in speech, swallowing, and respiratory function, often leading to premature mortality [21].

Therapeutic targeting of ACVR1 is complicated by its role in skeletogenesis, posing potential risks [22]. Nevertheless, several therapeutic approaches, including genetic therapies and small-molecule inhibitors, are currently under stringent clinical evaluation [23,24,25]. These therapies aim to modulate Activin A binding or inhibit ALK2 kinase activity. Other strategies involve modulating mTOR signaling and activating retinoic acid receptor gamma (RARγ) to inhibit chondrogenesis and endochondral ossification [24,25,26]. Notably, palovarotene, a RARγ agonist, was recently approved for FOP, though it has reported adverse effects [27,28].

This write-up seeks to synthesize the current knowledge of FOP, evaluate emerging therapies, and identify gaps to inform future research. It delves into the molecular structure underpinning the disorder, discusses current clinical management options, and critically evaluates the latest pharmaceutical advancements aimed at targeting this elusive condition.

## 2. A Brief Overview of the Disease FOP

The medical world’s awareness of FOP can be traced back to the 17th century, with early cases documented by Parisian physicians Guy Patin and André Falconet [29]. Patin, then Dean of the Faculty of Medicine at the University of Paris, detailed a case of progressive ossification of musculature along the spinal column, a seminal example of ectopic bone formation. Falconet described a woman whose body had turned “hard as wood”, an early observation of heterotopic ossification (HO).

Over the years, the condition accrued various nomenclatures including myositis ossificans progressiva, stone man syndrome, wood man syndrome, and Münchmeyer’s disease. Reports from 1938 introduced additional perspectives, detailing peculiar bone growths, e.g., “shoemaker femur” and “rifle shoulders”, which represented another form of ectopic bone formation [30]. The term “Fibrodysplasia Ossificans Progressiva” was officially adopted in the 1970s thanks to Dr. Victor McKusick of the Johns Hopkins University School of Medicine, who introduced the term to more accurately encompass the range of soft tissues, including tendons and ligaments, that could ossify [31]. 

FOP’s prevalence varies significantly based on geographic and ethnic differences [4,5,6,30,32,33,34]. North America reports the highest prevalence at 0.65 cases per million, followed by Western Europe at 0.47, Latin America at 0.27, Africa at 0.05, and the Asia–Pacific region at 0.04 [4,5]. Notably, Sweden, France, and Finland have higher prevalences of 1.43, 1.36, and 1.01, respectively, contrasted with Spain’s 0.36 per million [4,5,6,35]. The United States leads in registered FOP cases, accounting for 25.6%, followed by China at 10.8% and Brazil at 8.4% [6,30,32,33,34]. A striking 93% of individuals with FOP show symptoms by age 15, with symptom onset from 2.5 to 7 years and diagnosis from 4.8 to 10 years, varying by country [6,15,16,33,34,36]. Diagnosis typically occurs at a mean or median age of 4.8 to 10 years, varying by country [6,36]. However, cases exist where symptoms appear or diagnosis is made later in life, beyond the age of 25. Notably, the age of diagnosis in Asian countries, e.g., China and Japan, is often younger compared to Western countries [34,36]. Significant data gaps, especially in parts of Africa, the Mediterranean, and South Asia, highlight conspicuous disparities in healthcare access and awareness.

Clinically, FOP manifests in a systematic manner, with bilateral hallux valgus manifesting at birth and painful soft tissue flare-ups leading to HO [14,15,16,17,18,19]. These flare-ups may be triggered by various forms of trauma, e.g., surgery, injury, infection, or even a mild fever, and manifest as swelling, inflammation, and pain in the affected muscles, which subsequently ossify. However, the etiology of most flare-ups remains idiopathic. FOP’s pathophysiology involves complex interplay between aberrant inflammation and misplaced bone formation, leading to secondary symptoms, e.g., limb abnormalities, scoliosis, developmental hip dysplasia, and conductive hearing loss. FOP generally progresses in a cranial-to-caudal and axial-to-appendicular sequence, especially in adolescents and young adults. A significant complication of FOP is thoracic insufficiency, stemming from abnormal bone growth constraining chest and lung expansion, thereby precipitating respiratory challenges [37]. Moreover, while deteriorating pulmonary function affects over one-third of patients in late adolescence, all FOP individuals invariably confront lung complications as the disease approaches its terminal stage [15,16,33,34].

Diagnosing FOP remains a significant challenge, with patients often misdiagnosed with conditions like cancer and subjected to unnecessary, potentially harmful medical procedures. On average, accurate diagnosis occurs four years after the initial presentation of symptoms [38]. Despite advancements in both traditional and modern medicine, effective treatment options for FOP remain elusive. 

## 3. The Complex Molecular Tapestry of FOP

**Figure 1:**
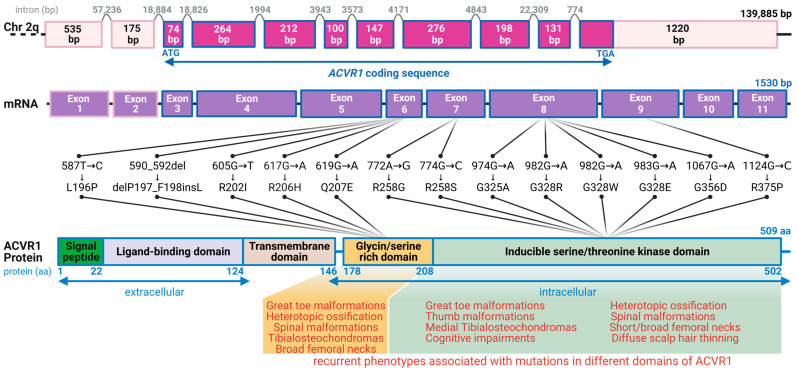
Schematic overview of the human *ACVR1* gene, its molecular architecture, and mutational landscape in FOP. This diagram depicts the structural components of the human *ACVR1* gene, localized on chromosome 2q24.1, which comprises 9 coding exons spanning 1530 base pairs (bp). The resulting encoded protein, ACVR1/ALK2, consists of 509 amino acids. Mutations linked to FOP predominantly affect the glycine/serine-rich and inducible serine/threonine kinase domains of the protein. Notably, the *ACVR1^R206H^* mutation is associated with >80% of reported FOP cases. Phenotypes commonly associated with mutations affecting different domains of ACVR1/ALK2 have been shown in [14,32,34,40,41,42]. While the mutations displayed in the figure are recurrent, they are not exhaustive. The lengths of the exons at the genomic level have been shown inside each box (Created with BioRender: NC263WVTV4).

Historically, the medical community’s understanding of FOP has been hindered by multiple technical and clinical obstacles [39]. These range from the challenge of obtaining viable tissue samples without exacerbating flare-ups to the absence of systematic data regarding early pathophysiology, which often lead to misdiagnoses. Moreover, the absence of reliable cellular and animal models, due to a lack of genetic insight, further hampered progress until the last two decades [2,7]. In a groundbreaking effort in FOP research, the collaborative work of Eileen M. Shore and Frederick Kaplan at the University of Pennsylvania in 2006 identified a heterozygous missense variant—ACVR1: 617G>A; R206H—in the *ACVR1* gene, a variant present in nearly all FOP cases [7,14,34,40,41,42]. Subsequent research identified additional pathologically relevant mutations within the *ACVR1* gene, mainly in regions encoding the protein’s intracellular domains, including, but not limited to, G328E, R258S, and G356D (Figure 1) [19,43,44]. While most mutations are spontaneous or de novo, a few multi-generational FOP patients display an autosomal dominant inheritance pattern [45]. It is important to note that not all missense mutations in the *ACVR1* gene are associated with FOP, as mutations in this gene have also been reported to be linked to congenital heart disease [46]. In addition, the presence of somatic mutations in *ACVR1* is reported in 1 out of 4 pediatric patients with diffuse intrinsic pontine glioma (DIPG), a brain tumor that occurs in children and affects the brainstem [47]. 

**Figure 2:**
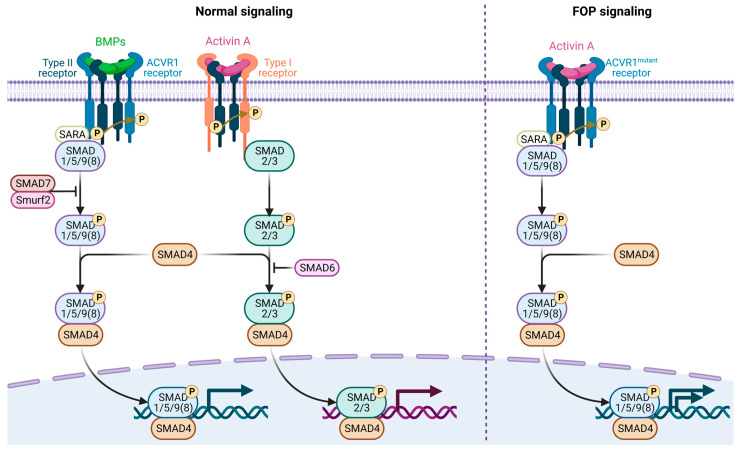
Canonical and aberrant BMP signaling and FOP. The canonical and aberrant activin A-mediated BMP signaling observed in FOP via mutant ALK2 receptors has been shown. Under physiological conditions, BMP or activin A ligands orchestrate the formation of a heterotetramaric receptor complex, comprising a homodimer of type II receptors in conjunction with a homodimer of type I receptors (e.g., ALK2 for BMP, ALK4/7 for activin A). This complex serves as a substrate for intramolecular phosphorylation events: the type II receptor phosphorylates the type I receptor, thereby activating it. Upon activation, the type I receptor, in turn, phosphorylates intracellular SMAD proteins—specifically SMAD1/5/9(8)—in the context of BMP signaling and SMAD2/3 in the context of activin A signaling. This series of phosphorylation events, at the presence of SMAD4, culminates in the effective transduction of canonical BMP and TGF-β signaling pathways, respectively. The pathological landscape of FOP is characterized by aberrant activin A signaling, which anomalously cross-activates BMP signaling via mutated ALK2 receptors. This deviation from the canonical pathway represents a critical molecular mechanism underlying the pathological manifestations of FOP. This mutant ALK2 receptor also renders the overall signaling hyperactive (Created with BioRender: BM25VJZ5RH).

From a genomic standpoint, the *ACVR1* gene, which encodes the Activin A receptor type I or activin receptor-like kinase-2 (ACVR1/ALK2), is located on the long arm of chromosome 2 [7]. This receptor plays a key role in the bone morphogenetic protein (BMP) signaling pathway, essential for developmental processes, such as bone and cartilage formation [48]. Under normal circumstances, secreted BMPs bind to complexes of type I and type II serine/threonine kinase BMP receptors on the cell surface, e.g., ACVR1 for BMP and ALK4/7 for activin A, setting off an intracellular signaling cascade [Figure 2] [13,22]. In the absence of BMP ligands, a regulatory protein called FKBP1A (FK506 binding protein 1A) binds to ACVR1, inhibiting its ability to bind effector molecules [19,44,49,50]. When BMP ligands are present, the type II receptor phosphorylates the type I receptor within its glycine/serine-rich domain [2,8,9,10,11,12,51,52,53,54]. This results in FKBP1A’s release, allowing ACVR1 to bind and phosphorylate intracellular BMP-responsive transcription factors known as receptor-regulated SMADs (R-SMADs), s SMAD1/5/9(8) [13]. The phosphorylated R-SMADs then form a complex with the co-mediator SMAD4. This complex then associates with co-activators or co-repressors to regulate transcription related to endochondral ossification. While SMAD1 and SMAD5 activate transcription in this context, SMAD9 serves as a transcriptional repressor.

In FOP, mutations like *ACVR1^R206H^* cause the receptor to become hyperactive, even when it should be in an inactive state [Figure 2] [2,55]. Initially, mutations in the *ACVR1* gene were believed to enhance BMP signaling through ACVR1/ALK2 complexes, thereby triggering heterotopic ossification in FOP. However, subsequent research has revealed a more complex molecular landscape [2]. The transforming growth factor-β (TGF-β) superfamily has emerged as a crucial player in the molecular etiology of both FOP and trauma-induced HO. Key ligands in this superfamily, e.g., TGF-β1 and BMP, activate R-SMADs and participate in non-canonical pathways involving molecules like mitogen-activated protein kinases (MAPKs), phosphoinositide 3-kinases (PI3K), protein kinase B (PKB/AKT), mechanistic target of rapamycin (mTOR), and TGF-β-activated kinase 1 (TAK1) [2,12,13,56]. Mutation in *ACVR1* causes ACVR1 to remain active without ligands, disrupting cellular homeostasis and causing the pathological ossification of connective and muscle tissues [57]. The mutated receptor also shows altered affinity toward its regulatory protein FKBP1A, impairing proper receptor regulation and leading to abnormal bone and cartilage growth, ultimately resulting in joint fusion [2,12,13]. Furthermore, *ACVR1^R206H^* makes ALK2 hypersensitive to Activin A, a phenomenon referred to as neo-receptorization [58]. Under normal homeostatic conditions, ACVR1 modulates BMP signaling to maintain cellular stability. However, the *ACVR1^R206H^* mutation disrupts this equilibrium, causing the receptor to advocate for increased BMP signaling, leading to pathological outcomes.

While our understanding of FOP’s pathobiology has significantly advanced, several key questions remain unanswered, particularly concerning the role of ACVR1 receptor complexes in normal cellular homeostasis and the pathways influencing fibro-adipogenic progenitors (FAPs) toward osteogenic phenotypes under traumatic or inflammatory conditions [39,59]. Additionally, there is a compelling need to elucidate the unique structural perturbations brought about by *ACVR1^R206H^*, including its propensity for ACVR1 homodimerization.

Targeting this complex’s etiological framework has proved therapeutically elusive. Antibodies against Activin A have demonstrated some efficacy in mitigating HO in animal models; however, counterintuitively, anti-ACVR1 antibodies designed to neutralize receptor function have exacerbated HO, thereby complicating treatment paradigms [12,23,25,59,60]. This seemingly paradoxical observation can plausibly be explained through understanding ACVR1’s role in the BMP signaling pathway. ACVR1 serves as a receptor for BMPs, known promoters of osteogenesis. In FOP, the mutant ACVR1 receptor remains constitutively active, culminating in inappropriate bone formation. Instead of blocking this process, anti-ACVR1 antibodies might stabilize the active receptor or trigger receptor clustering, intensifying HO. Current avenues of investigation include Activin A antibodies, mTOR inhibitors like rapamycin, and retinoic acid receptor-gamma (RARγ) agonists, which are collectively poised to revolutionize our understanding of FOP and potentially provide clinically viable interventions. Also, genetic therapeutic approaches, e.g., gene therapy, and allele-specific gene knockdown, are being explored [61,62,63,64,65]. 

Thanks to the extraordinary research conducted in the past two decades, our understanding of FOP has transitioned from a simplistic, reductionist model to a complex interplay between signaling cascades and regulatory molecules [39,57]. Navigating this complex terrain remains a daunting task, necessitating sustained, multidisciplinary research efforts to develop targeted, effective therapies for this debilitating condition. 

## 4. Clinical Presentation, Diagnosis, and Management of FOP

Accurate and timely identification of FOP is crucial [33,66]. Pediatricians are often the first medical professionals to encounter children with FOP, making their awareness of the disease essential for early diagnosis and appropriate management. Diagnostic suspicion is vital, since a lack of it can lead to delays and potentially harmful misdiagnoses, which may result in invasive and counterproductive testing like biopsies [66,67]. These procedures could exacerbate the condition by inducing flare-ups and promoting HO. 

Clinically, FOP is often initially suspected based on the presence of hallmark congenital deformities, predominantly malformations of the great toes, e.g., hallux valgus and macrodactyly [38,68]. The onset of episodic flare-ups, marked by painful and warm soft tissue swellings, usually begins in the first decade of life [17]. Triggered by a range of pro-inflammatory factors, these flare-ups result from underlying inflammation in the tendons, ligaments, or muscles. Some studies have identified brain abnormalities in FOP patients, including hamartomas and dysmorphisms in the brainstem, alongside signal abnormalities or calcifications in the dentate nucleus and basal ganglia, linking *ACVR1* mutations to potential disruptions in normal brain development and function [69]. Moreover, a subset of patients may manifest non-standard features, referred to as FOP-plus (FOP^+^), which can include a range of anomalies, varying from tibial osteochondromas to cognitive impairment [14]. These features are often associated with specific *ACVR1* mutations, e.g., R206H and Q207E. The neurological phenotypes sporadically observed in FOP and FOP*^+^*-phenotypes may be attributed to the impact of mutant *ACVR1* on the nervous system, a view further supported by the involvement of *ACVR1* mutations in DIPG [47,69]. 

FOP frequently manifests its effects on the cervical spine at an early stage [70]. This involvement begins with localized flare-ups and neck stiffness, which may progress to bony ankylosis, severely restricting neck movement. FOP can affect various body joints in an unpredictable manner, often leaving patients wheelchair-bound by their late teens or early twenties. Additional complications can include hearing loss, malnourishment due to jaw involvement, and life-threatening thoracic insufficiency syndrome. Although clinical presentation is significant, the gold standard for diagnosing FOP is using DNA sequencing to identify underlying mutations in the *ACVR1* gene. Biopsy and other invasive procedures should be avoided, as they risk exacerbating the condition by promoting HO [71].

Current clinical management of FOP is primarily aimed at controlling inflammation, as it triggers the cascade of events leading to HO [25,26,32]. However, managing triggering events remains challenging, as they can range from significant physical trauma to seemingly trivial incidents. The pharmacological landscape is evolving, categorized into three classes: Class I medications like high-dose corticosteroids, non-steroidal anti-inflammatory drugs (NSAIDs), cyclo-oxygenase 2 (COX2) inhibitors, mast cell inhibitors, aminobisphosphonates, and muscle relaxants are used to manage flare-ups; Class II medications have theoretical but unproven applications in FOP; and Class III medications are under clinical investigation [32,55,72,73,74,75,76,77,78,79]. 

Flare-ups are usually managed through a 3–5-day course of high-dose corticosteroids, e.g., prednisone, to alleviate inflammation and tissue edema. Patients with FOP are often able to recognize the signs of an impending flare-up and may initiate communication with their physician to start prednisone treatment. However, while effective, corticosteroids are not a cure for FOP and have numerous limitations and potential side effects, including osteoporosis, diabetes, hypertension, infections, weight gain, mood changes, and adrenal insufficiency [80]. Additionally, frequent use of corticosteroids to manage swelling in the trunk and neck is not recommended due to difficulties in monitoring flare-up onset. As a result, corticosteroids should be used cautiously, under medical supervision, and typically for short durations. After discontinuing corticosteroids, mast cell inhibitors, aminobisphosphonates, NSAIDs, and COX-2 inhibitors can be used to manage subsequent flare-ups [25,55,80,81,82]. Small doses of a muscle relaxant may help to relieve muscle spasms [55,80,81]. 

Bisphosphonates, e.g., aminobisphosphonates, a class of drugs commonly used to treat osteoporosis, can potentially reduce pain, inflammation, and tissue edema during flare-ups and may even prevent or delay heterotopic ossification in FOP patients [72,80,83,84]. These drugs work by inhibiting osteoclast activity, which is responsible for bone breakdown [83]. There are also several risks associated with bisphosphonates, e.g., gastrointestinal irritation, renal impairment, and osteonecrosis of the jaw, suggesting that the use of bisphosphonates for FOP needs to be cautious [72,85]. 

Palovarotene, a selective retinoic acid receptor gamma agonist, has been repurposed for the treatment of FOP [86]. Researchers led by Maurizio Pacifici at the Children’s Hospital of Philadelphia conducted foundational work, and Ipsen Biopharmaceuticals later licensed the drug [87,88]. It received FDA approval for medical use in FOP in 2023, after previously receiving approval from Health Canada in 2022 [27,28]. By binding to RARγ, an essential regulator of skeletal development, palovarotene activates the retinoic acid signaling pathway, which employs the same SMAD proteins used via the ACVR1-dependent BMP pathway [28,65]. This allows palovarotene to interfere with aberrant BMP signaling, thereby reducing the risk of HO. Despite its promise, Palovarotene faced challenges in clinical trials, including a failed futility test, and raised safety concerns [86,89]. Palovarotene was presumed to be associated with the risk of early growth plate closure in pediatric patients, impacting their height and development [90]. The drug modulates ACVR1’s downstream signaling without directly targeting it; therefore, it does not differentiate between wild-type and mutant ACVR1 [28]. Moreover, significant adverse effects have been reported, including dry skin, lip dryness, arthralgia, pruritis, pain in extremity, rash, alopecia, erythema, headache, back pain, skin exfoliation, nausea, musculoskeletal pain, myalgia, dry eye, hypersensitivity, peripheral edema, and fatigue [26,91]. Additional concerns include potential embryotoxic and teratogenic effects and associations with depression, anxiety, mood alterations, and suicidal thoughts [27,91]. All these issues have led to contraindications of palovarotene for a significant portion of the FOP population, particularly in pregnancy and epiphyseal closure in pediatric patients [27,91].

Experimental approaches for FOP, including allele-selective nucleic acid-based RNA knockdown strategies, BMP receptor kinase inhibitors, gene therapy, stem cell therapies, and immuno-therapies, hold promise for both managing symptoms and modifying the course of FOP [24,25,92]. Overall, the pharmacological landscape for FOP is evolving, with the goal of achieving a balance between symptom management and disease modification. In the subsequent sections of this write-up, we will focus on these experimental approaches that are currently being investigated to effectively treat FOP. 

## 5. Animal Models of FOP

The development and characterization of animal models have been paramount in delineating the pathogenesis of FOP and expediting the discovery of therapeutic interventions. These models have provided invaluable insights into the molecular mechanisms underlying the disease and facilitated the evaluation of potential therapeutic strategies. 

The *ACVR1^Q207D^* mouse, developed by Tomokazu Fukuda’s group at the National Institute of Environmental Health Sciences, NC, marked a significant milestone in FOP research [93]. Localized and global *ACVR1^Q207D^* expression models have provided insights into the role of inflammation and injury in the process of HO [93,94]. Notably, the global model revealed that *ACVR1^Q207D^* expression alone was insufficient for HO formation, underscoring the necessity of secondary insults. Although Q207D mutation in *ACVR1* does not occur naturally in human FOP patients, it confers constitutive activation of the ACVR1 receptor, doing so in a manner similar to, but more severe than, the FOP-associated mutations R206H and Q207E [95,96]. This model has also been instrumental in elucidating the molecular underpinnings of FOP, affirming the necessity of Type II receptor complex formation for ligand-independent BMP signal transduction and facilitating the in vivo evaluation of therapeutics, e.g., palovarotene [87,97].

Complementing the *ACVR1^Q207D^* mouse, the *ACVR1^R206H^* models further refined our understanding of FOP’s molecular pathology [98]. These models recapitulated key FOP features, including classic hind limb digit malformation and HO development, thereby offering a clinically relevant platform for mechanistic studies and therapeutic evaluation [99]. Notably, the conditional *ACVR1^R206H^* mouse model elucidated the role of activin A in aberrant *ACVR1^R206H^* activation and provided a framework for the investigation of targeted interventions, e.g., activin A-blocking antibodies [57].

Another conditional mouse model, the doxycycline-inducible FOP-ACVR1(R206H), was developed by the groups of Makoto Ikeya and Yasuhiro Yamada at Kyoto University [100]. This model utilizes a conditional transgenic expression system to circumvent the perinatal lethality frequently observed in previous models. In this model, human FOP-ACVR1 (R206H) is inserted into the collagen type I alpha 1 (*Col1a1*) locus, allowing expression predominantly in differentiated osteoblasts and, therefore, significant expression in bones [100,101]. While *Col1a1* is expressed in various tissues and, theoretically, the newly inserted *ACVR1^R206H^* should be detectable in all tissues, no expression was found in skeletal muscle, indicating a preference for expression patterns. Unfortunately, specific data regarding the expression efficiency of FOP-ACVR1 in muscle and cartilage tissues are not available, perhaps due to considerable background signals in these tissues.

To ascertain the progenitor cell populations driving HO, researchers have employed both *ACVR1*-mediated and non-*ACVR1*-mediated mouse models, identifying contributions from various progenitor cell populations, including Tie2*^+^* endothelial cells, circulating osteogenic progenitor cells, and Scx*^+^* cells [102,103,104,105]. These findings underscore the complexity of the HO process and spotlight the importance of delineating the interplay between different progenitor cells in FOP pathology.

Beyond murine models, the embryonic chicken and zebrafish models have provided valuable insights into the effects of *ACVR1* mutations on early limb development and dorsoventral axis establishment, respectively [51,95,106,107,108]. Furthermore, the novel adult zebrafish model circumvents the lethality associated with the embryonic expression of constitutively active *Acvr1l*, offering a promising avenue for studying FOP in a mature organismal context [109].

In addition to the aforementioned models, the fruit fly (*Drosophila melanogaster*) has been an invaluable model for FOP studies. Leveraging the high degree of conservation between human and *Drosophila* BMP signaling pathways, researchers have successfully employed fruit fly models to probe the intricacies of *ACVR1*-mediated signaling. The utility of the fruit fly model was exemplified by studies that employed the *Drosophila* orthologs of *ACVR1*, namely saxophone (*sax*) and thickveins (*tkv*), to interrogate the molecular consequences of FOP-associated mutations [110]. Through these investigations, researchers were able to delineate the perturbations in BMP signaling elicited by *ACVR1* mutations, furthering our understanding of FOP pathogenesis at the molecular level. Moreover, the fruit fly model offers a high-throughput platform for genetic and pharmacological screens, thereby accelerating the identification of potential therapeutic agents.

Collectively, these animal models serve as an indispensable arsenal in the battle against FOP, facilitating a comprehensive understanding of the disease’s molecular underpinnings, revealing novel therapeutic targets, and providing a robust platform for the assessment of potential interventions. The synergy of insights learned from these models undoubtedly propels this field towards the ultimate goal of devising effective treatments for FOP.

## 6. Experimental and Prospective Therapeutic Approaches for FOP

**Figure 3:**
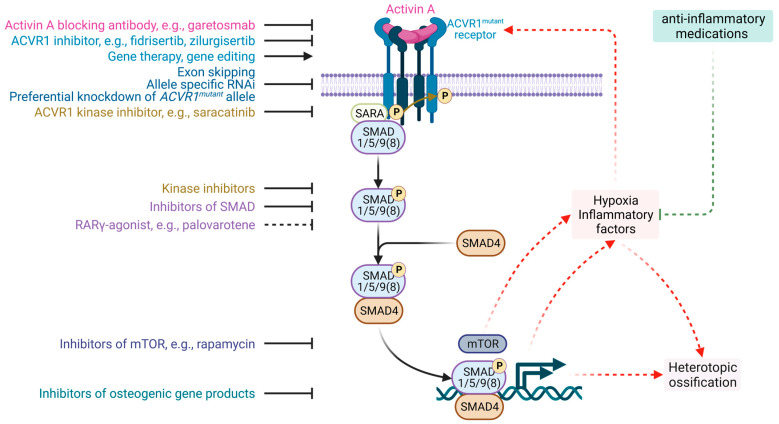
Targeting Aberrant BMP Signaling in FOP. This figure illustrates the aberrant BMP signaling pathway in FOP, identifying specific points where therapeutic drugs and strategies intervene to modulate the pathway. Activin A blocking antibodies, e.g., garetosmab, inhibit activin A-mediated signaling. ACVR1 inhibitors, including fidrisertib and zilurgisertib, inhibit the activity of mutant ACVR1 receptors. Exon skipping, allele-specific RNAi, and preferential inducers of *ACVR1^mutant^* alleles target the genetic level to reduce the expression of mutant ACVR1 receptors. ACVR1 kinase inhibitors, e.g., saracatinib, inhibit the kinase activity of the ACVR1 receptor. SMAD kinase inhibitors and inhibitors of SMAD disrupt the downstream signaling of the BMP pathway. RARγ-agonists, e.g., palovarotene, enhance the proteasome degradation of SMAD1/5/9(8) proteins. Inhibitors of mTOR, e.g., rapamycin, and inhibitors of osteogenic gene products affect the cellular responses to BMP signaling. Additionally, gene therapy and gene editing are identified as novel strategies that can be employed to directly target and correct the genetic mutations associated with FOP. Anti-inflammatory medications can reduce the risk of HO in FOP patients by inhibiting inflammatory factors. (Created with BioRender: UO26177N1O).

Numerous experimental and prospective approaches are emerging that directly or indirectly target various components of the aberrant BMP pathway or the broader FOP pathology [Figure 3]. As we transition towards translational medicine, these innovative strategies provide hope of overcoming the limitations of traditional pharmacotherapies, each bringing its own set of promises and challenges [Figure 3]. These can be grouped into four main categories: genetic approaches, enzymatic and transcriptional target modulators, stem cell therapies, and immunotherapies [24,25,92]. In addition, there have been some continuous works aiming to repurpose drugs for FOP [111]. Recent efforts, e.g., Shaikh et al., 2023 [24], have listed the emerging therapeutic strategies for FOP. In this section, we aim to provide a holistic review of the currently developing and recently emerging therapies and provide unique insights into the future of FOP therapeutic research. 

CRISPR-Cas (Clustered Regularly Interspaced Short Palindromic Repeats and CRISPR-associated proteins) technology has captured significant attention for its precise ability to alter the mutant *ACVR1* gene [24]. This precision is a marked departure from small-molecule inhibitors, which, while effective, often carry systemic side effects like skin and metabolic issues. Another promising avenue is RNA-based therapies designed to specifically silence the mutant *ACVR1* gene [63,64,65]. This could halt the abnormal bone formation characteristic of FOP, offering a targeted therapeutic approach. In parallel, researchers are investigating crucial enzymatic pathways, e.g., glycogen synthase kinase-3 (GSK-3β) and transcription factors like peroxisome proliferator-activated receptor-γ (PPARγ). Both play significant roles in bone formation and inflammation [24,25,72,87]. Small molecule inhibitors targeting these proteins offer an alternative that could avoid some of the risks associated with gene therapies. Moreover, innovative strategies employing mesenchymal stem cells (MSCs) and induced pluripotent stem cells (iPSCs) are being explored for their potential in tissue regeneration [24]. These strategies aim to differentiate stem cells into bone-forming cells to replace damaged tissues. However, questions remain about how these cells behave in the body and their potential to exacerbate FOP symptoms. In an era when immunotherapy continues to revolutionize cancer treatment, its application for treating FOP is intriguing [24]. Using tools like monoclonal antibodies and immune checkpoint inhibitors could represent a significant shift in FOP treatment, potentially moderating the heightened immune responses often seen in patients with this condition.

Despite these advances, challenges remain. While there are animal models available for testing, they often do not fully replicate the human condition of FOP and/or have other limitations [98]. Additionally, there is the risk of disease flare-ups post-treatment and the variable manifestations of the disease. Each of these hurdles necessitates rigorous preclinical and clinical evaluations. The emerging therapies are expanding the treatment landscape for FOP and enhancing our understanding of both bone biology and immune responses. 

### 6.1. Genetic Therapeutics for FOP

The advent of genetic therapies presents a promising frontier in FOP therapeutic research. These therapeutic strategies primarily focus on the genetic root of the condition, typically mutations in the *ACVR1* gene. Genetic approaches, e.g., gene editing, gene addition, gene silencing, and gene replacement, are currently being explored [Table 1] [92]. Each approach offers a unique set of advantages and challenges; however, collectively, they aim to rectify or modulate the expression of the mutated gene, thereby alleviating the clinical outcome. These genetic therapies could mark a paradigm shift in the treatment of FOP, offering not only symptomatic relief but also, potentially, a long-term solution.

#### 6.1.1. CRISPR-Cas Gene Editing Therapies

CRISPR-Cas is a genome-editing tool that allows precise targeting and modification of DNA sequences [112,113]. Lately, CRISPR-Cas9 has captivated the scientific community with its groundbreaking capabilities in gene editing [114]. In the context of FOP, CRISPR can be bioengineered to target the mutant *ACVR1* allele and modify or correct the mutation, thereby facilitating the translation of a corrected version of ACVR1 [Figure 3] [115]. The delivery of the CRISPR-Cas9 complex is generally facilitated through viral vectors, e.g., adeno-associated virus (AAV), or lipid nanoparticles (LNPs), with tissue specificity ensured through vector selection [116,117]. A carefully chosen vector, specific to the tissue of interest, ensures that gene editing only occurs in targeted cells. Although the technology is admired for its unparalleled precision and resultant genomic permanence, significant issues, including off-target mutations, ethical considerations, and the complex process of regulatory approvals, pose challenges to the clinical adoption of CRISPR-Cas gene editing therapies for FOP. 

#### 6.1.2. RNA-Based Therapies

RNA-based therapies employ antisense oligonucleotides (ASOs), e.g., small interfering RNAs (siRNA), gapmers, aptamers, agomirs and antagomirs, and steric-blocking oligonucleotides—these ASOs temper gene expression post-transcriptionally with high specificity through a wide array of mechanisms [118]. An allele-specific silencing strategy is particularly appealing for FOP, as it offers the possibility of specifically targeting and knocking down the mutant *ACVR1* allele, leaving the wild-type *ACVR1* allele untouched, thus ensuring that only the wild-type allele is translated [Figure 3] [119]. Studies by Kaplan et al. and Takahashi et al. demonstrated the development of allele-specific siRNAs, while Maruyama et al. has recently explored locked nucleic acid (LNA) gapmers for targeted suppression [63,64,65]. Gapmers, reported recently by Maruyama et al. from our laboratory, preferentially lowered *ACVR1^R206H^* expression and the level of the protein encoded while leaving most of the normal products intact, leading to the suppression of osteogenic differentiation in vitro [63]. Despite their high specificity and reversible action, RNA-based therapies may necessitate frequent administration. In addition, the in vivo delivery of these RNA-based therapies poses significant challenges for their widespread clinical use.

#### 6.1.3. Gene Therapies

Drawing parallels from the therapeutic success in treating monogenic disorders, e.g., lipoprotein lipase deficiency, inherited retinal dystrophy, and spinal muscular atrophy, it is conceivable that extant gene therapies could be efficacious for FOP, as it is also caused by a monogenic gain-of-function mutation in *ACVR1* gene [92,120,121]. In general, gene addition aims to introduce genes encoding missing proteins or corrective proteins if a genetic mutation produces defective proteins [Figure 3]. A landmark study by Yang et al. has exemplified the potential of gene replacement therapy [62]. Using an AAV vector, the researchers simultaneously silence the mutated gene (*ACVR1^R206H^*) and reintroduce its wild-type variant. This rescued the aberrant BMP signaling pathways and effectively prevented and treated trauma-induced HO in a murine model of FOP. These findings were substantiated by a prior proof-of-concept study by the same researchers, wherein a recombinant AAV vector carrying a healthy *ACVR1* gene, coupled with artificial microRNA to silence the mutant gene, suppressed ectopic bone formation in mice [61]. While the prospective benefits of gene therapies are considerable, the challenges are non-trivial. Notably, the looming risks of insertional mutagenesis and immunological responses against viral vectors warrant caution.

#### 6.1.4. Future Prospects for Genetic Approaches for FOP

When comparing these therapeutic approaches, the intricate technical variables, e.g., the choice between in vivo and ex vivo interventions, as well as the accessibility of targeted tissues, must be taken into consideration [23]. From an ethical and regulatory standpoint, all these approaches encounter difficulties. Nevertheless, advancements in nanoparticle delivery systems, improvements in CRISPR specificity, and innovations in RNA formulations offer a promising outlook for the future of FOP therapeutics.

### 6.2. Enzymatic and Transcriptional Target Modulators

The treatment landscape for FOP is gradually being reshaped by enzymatic and transcriptional target modulators, which aim to attenuate or reverse the pathology by selectively targeting key signaling pathways and gene expression mechanisms [122]. This subsection describes the principal types of modulators under investigation and their therapeutic potential, supported by preclinical studies and ongoing research. To realize the full potential of these modulators in clinical practice, it is imperative to improve our technical prowess in predicting long-term effects and surmounting challenges, e.g., off-target effects and therapeutic resistance through rigorous clinical trials and sustained preclinical research. 

#### 6.2.1. Targeting BMP Signaling: Antagonists and Allosteric Modulators

The BMP signaling cascade is central to the pathogenesis of FOP. Transcriptional modulators, including BMP receptor antagonists, operate by preventing the ligand-induced conformational change required for intracellular signaling [122]. Allosteric inhibitors e.g., dorsomorphin target BMP type I receptors, particularly ACVR1, have shown efficacy in abrogating ectopic ossification in animal models. Ligand traps, synthesized as chimeric proteins comprising the extracellular domain of ACVR2B or ACVR2A fused to the Fc region of IgG1, have also demonstrated their efficacy in sequestering BMP ligands [60,123]. Palovarotene, the only FDA-approved therapy for FOP, indirectly abrogates BMP signaling, making it a leading candidate for further investigation [Figure 3] [27,28]. Other proposed approaches include BMP receptor kinase inhibitors, e.g., dorsomorphin, downstream BMP signaling inhibitors such as fendiline and perhexiline, and fungal metabolite osteoblast differentiation inhibitors such as NG-391, NG-393, and trichocyalide A/B [55,124,125,126,127].

#### 6.2.2. Dual-Targeting via mTOR Pathway Inhibition

The mTOR signaling pathway plays a multifaceted role in FOP, particularly in osteogenic and chondrogenic differentiation [100]. Rapamycin, a well-known immunosuppressive drug, exerts its effects through the inhibition of mTORC1, thereby thwarting activin A-induced chondrogenesis and osteogenesis [Figure 3]. The inhibition of mTORC1 using rapamycin, in turn, mitigates activin A-induced chondrogenesis and osteogenesis, presenting a compelling avenue for FOP intervention [26]. Besides rapamycin, another promising avenue in FOP treatment revolves around PI3Kα inhibitors, notably BYL719 [128]. These inhibitors have exhibited considerable potential in preclinical models, especially in cells harboring *ACVR1* mutations associated with FOP. Their mechanism of action encompasses the simultaneous inhibition of key signaling pathways, including SMAD and AKT, aside from the mTOR pathway [26,122]. This multifaceted approach underscores their promise to effectively manage FOP.

#### 6.2.3. Neutralizing Hyperactivated Activin A Signaling via Antibody Modulation

Activin A signaling, being pathologically upregulated in cells with the *ACVR1^R206H^* mutation, has been efficaciously modulated using neutralizing antibodies. Such antibodies act to inhibit the ligand–receptor interaction, thereby subverting downstream signaling and, ultimately, reducing heterotopic ossification. Currently, clinical trials are underway involving garetosmab, also known as REGN2477, which is a fully human IgG4 monoclonal antibody meticulously designed to specifically impede activin A [Figure 3] [57,129]. The primary objective of this trial is to rigorously evaluate the long-term safety profile and therapeutic potency of this innovative approach.

#### 6.2.4. Other Approaches: GSK-3β Inhibition and PPARγ Activation

Emerging candidates include GSK-3β inhibitors, which act as downstream effectors in BMP signaling, and PPARγ agonists, which preferentially induce adipogenesis over osteogenesis [126]. The former aims to block the osteogenic differentiation of pluripotent stem cells, while the latter counteracts osteogenesis through the upregulation of adipogenic genes.

#### 6.2.5. Challenges and Future Directions

While these pharmacological interventions offer promising avenues for FOP treatment, their inherent complexities, e.g., off-target effects and the specter of therapeutic resistance, must be judiciously considered [59,130]. The potential synergistic benefits of these multi-targeted approaches may present enhanced therapeutic efficacy, though this requires thorough combinatorial analysis to ascertain potential antagonistic interactions. Enzymatic and transcriptional modulators are at the forefront of therapeutic innovation for FOP [25,60,122]. However, a complete understanding of their long-term safety profiles, pharmacokinetics, and pharmacodynamics is imperative to facilitate the translation of these therapeutic modalities from bench to bedside. Additionally, comprehensive preclinical and clinical studies are imperative to determine the optimal dosing and scheduling for administration. The development of more specific modulators and conducting thorough preclinical studies to identify potential resistance mechanisms are pivotal to circumvent the risks of off-target effects and therapeutic resistance.

### 6.3. Stem Cell Therapies for FOP

The emergence of stem cell technologies, particularly MSCs and iPSCs, has offered new vistas of therapeutic possibilities [131,132]. MSCs, multipotent stromal cells derived from tissues, e.g., bone marrow and adipose tissue, have shown potential in mitigating abnormal bone growth in FOP models through their ability to differentiate into osteoblasts, chondrocytes, and adipocytes. On the other hand, iPSCs, derived from adult cells reprogrammed into an embryonic-like pluripotent state, proffer the possibility of creating disease-specific cellular models, thereby facilitating tailored therapeutic interventions [131,132,133,134]. While both technologies are in the experimental stage, their ability to potentially replace malfunctioning cells and offer insights into the disease’s mechanisms holds significant promise for the future management of FOP. Addressing challenges, e.g., inconsistent outcomes, potential tumorigenesis, and possible immune reactions, necessitates rigorous clinical trials, the ongoing monitoring of long-term effects, and the refinement of technologies to ensure safety and efficacy.

#### 6.3.1. Mesenchymal Stem Cells (MSCs)

Mesenchymal stem cells (MSCs), multipotent stromal cells derived from various tissues including bone marrow and adipose tissue, can transform into various tissue types, including bone and cartilage. These cells are considered to be excellent candidates for replacing the malfunctioning cells in FOP [131]. Researchers are also exploring genetically modifying these MSCs to improve their effectiveness, targeting specific genes or cellular pathways contributing to the disease. However, the use of MSCs has its own set of challenges. Studies show that there are several inherent concerns associated with MSC therapies, which include administration site reactions, the ability of cells to move from placement sites and change in inappropriate cell types and/or multiply, the inconsistency of cells in producing expected outcomes, and the likelihood of developing tumors [135,136]. Furthermore, since MSCs come from external sources, the body’s immune system might react against them. Rigorous preclinical studies, followed by meticulously designed clinical investigations, are imperative to elucidate the safety and efficacy of MSCs in patients with FOP. In addition, the longitudinal impacts of MSC transplantation need to be thoroughly evaluated before MSC therapies can enter the clinic for FOP. Strategies including genetic engineering and efficient stock-still placement can be employed to attenuate the risk of tumor development and undesired cell movement, thereby enhancing the safety profile of MSCs.

#### 6.3.2. Induced Pluripotent Stem Cells (iPSCs)

Induced pluripotent stem cells (iPSCs), which are derived from adult cells reprogrammed into an embryonic-like pluripotent state, have the potential to transform into any cell type and can be tailored to the individual patient. These cells avoid the ethical concerns associated with using embryonic cells [133,134]. These reprogrammed cells can theoretically replace the dysfunctional bone-forming cells in FOP patients. The potential to develop patient-specific or disease-specific cells makes iPSCs incredibly useful for not just for treatment but also for research into FOP. Yet, iPSCs come with their own set of challenges. The process of reprogramming these cells is technically complex and expensive. Like MSCs, iPSCs also carry a small but significant risk of forming tumors. Similar to MSCs, extensive studies are needed to evaluate the safety and efficacy of iPSCs in FOP patients. The trials should assess the long-term effects of iPSC transplantation. The challenges include the technical complexity and cost of reprogramming cells. These can be addressed by developing more efficient reprogramming methods and finding ways to reduce costs.

#### 6.3.3. Challenges and Future Directions

Both MSCs and iPSCs offer promising new pathways for treating FOP [131,133,134]. Each has unique benefits but also comes with its own set of limitations. The next crucial steps involve refining these promising technologies and testing their safety and effectiveness in large, rigorous clinical trials. In this ongoing journey to find a definitive treatment for FOP, MSCs and iPSCs stand out as particularly promising candidates, having the potential to significantly change how this devastating disease is managed in the future.

### 6.4. Immunotherapy

Contemporary research is reconceptualizing FOP through an immunological lens, pointing toward an ensemble of immunological actors, including monocytes, macrophages, mast cells, and various cytokines that orchestrate pathological ossification [122,137]. Monoclonal antibodies and immune checkpoint inhibitors emerge as potential therapeutic strategies [60,122,138]. The transition of these strategies from bench to bedside mandates addressing challenges, e.g., ensuring specificity, minimizing side effects, and conducting extensive clinical trials, to establish long-term safety and effectiveness.

#### 6.4.1. Targeting Specific Antigens Using Monoclonal Antibodies

Monoclonal antibodies (mAbs) have emerged as biological pharmaceuticals with unparalleled specificity, demonstrating potential as a therapeutic strategy for FOP [60,122]. These are immunoglobulins meticulously engineered to bind to specific antigens expressed on the surfaces of errant cells, thereby marking them for immune-mediated destruction. The conjugation of monoclonal antibodies with cytotoxic agents, e.g., toxins or radioisotopes, offers an additional mechanism to selectively target and annihilate the cells responsible for HO. Monoclonal antibodies can be designed to target the complex signaling pathways that contribute to FOP, including aberrant BMP signaling and heightened cytokine production (IL-3, IL-7, IL-8, and IL-10) [139,140]. A comprehensive suite of preclinical and clinical studies is necessary to portray the safety profile and therapeutic efficacy of mAbs in the context of FOP. These studies also need to be carefully structured to ascertain the optimal dosage and administration schedule. The emergence of resistance to mAbs and their associated high costs represent significant hurdles. The development of combination therapies to preclude resistance, alongside the exploration of methodologies to reduce production expenses, are needed to make this strategy viable.

#### 6.4.2. Modulating Immune Responses Using Immune Checkpoint Inhibitors

Concurrent with the mAbs paradigm, immune checkpoint inhibitors (ICIs) stand as another modality under rigorous investigation for the treatment of FOP [138]. ICIs act by negating the downregulatory signals that often shield pathological cells from immune detection. In the context of FOP, where aberrant immune signaling contributes to pathogenesis, ICIs can modulate the immunological landscape, allowing more effective targeting of cells contributing to HO. Their ability to silence the immune system’s signaling pathways has implications for reducing the activation of key players like TGF-beta, NF-κB, and MAPK signaling. To propel ICIs into the clinical stage for the treatment of FOP, exhaustive preclinical evaluations are required to confirm their safety and effectiveness. Drawing parallels from oncology, where ICIs have ushered in a paradigm shift in therapeutic strategies, meticulously designed clinical trials are critical. Challenges inherent to this approach include off-target effects and immune-related adverse events analogous to those witnessed in oncology patients administered with ICIs [141]. Developing exquisitely specific ICIs and stringent evaluations could possibly ameliorate these challenges [142].

#### 6.4.3. Cellular Infiltrates

Inflammatory lesions in FOP often exhibit an accumulation of monocytes, macrophages, and mast cells, which seem to play pivotal roles in enhancing the inflammatory immune response [23,56]. These cellular infiltrates are responsible for the increased production of cytokines and chemokines, e.g., IL-3, IL-7, IL-8, IL-10, CCL5, CCR7, and CXCL10 [23,56,122]. Studies utilizing mouse models have illustrated that the depletion of macrophages and mast cells significantly mitigates heterotopic ossification, offering another potential avenue for treatment. Extensive preclinical and clinical studies are crucial to appraise the effectiveness of targeting cellular infiltrates as a therapeutic strategy for FOP. These studies must be formulated to determine if the attenuation of macrophages and mast cells can curtail HO. A conceivable challenge is the potential for adverse effects stemming from the depletion of these immune cells. Mitigation strategies include vigilant surveillance and the development of strategies to minimize potential adverse events, like those being developed in oncology.

#### 6.4.4. Prospective Immunotherapeutic Strategies for FOP

Immunotherapy offers a groundbreaking approach to FOP by capitalizing on the immunological peculiarities that drive its pathogenesis [23]. The specificity and targeted action of monoclonal antibodies, coupled with the immune modulation offered by checkpoint inhibitors, signify a potential therapeutic renaissance for this complex disease. Further study is warranted to investigate combination therapies and ascertain long-term safety and efficacy. In a disease state as devastating and complex as FOP, the advent of immunotherapy may represent an incremental advance and a quantum leap in our therapeutic armamentarium.

### 6.5. Repurposed Drugs for FOP: A Glimpse of Promise

Developing new medications for ultra-rare conditions like FOP is highly challenging for many reasons [39,143]. An alternative is to use existing drugs, known to be safe, for new purposes. This strategy, called drug repurposing, is faster, more cost-effective, and less risky [144]. It is especially useful for rare diseases like FOP, which face unique challenges in drug development [111,143]. Historically, many drugs, e.g., sildenafil and thalidomide, have found new uses through repurposing [143]. Currently, drugs like corticosteroids, celecoxib, and inhibitors targeting hypoxia-inducible factor 1α (HIF1α) and PI3Kα pathways have the potential to treat FOP by addressing its key pathways in FOP pathology, e.g., inflammation and BMP signaling, and they have the potential to inhibit HO [111]. Saracatinib, initially developed for cancers, has exhibited selective inhibition of ACVR1 [Figure 3] [145]. In preclinical models, it effectively prevented HO, thereby emerging as a promising candidate for further clinical trials. 

While the findings of initial and/or preliminary clinical studies of repurposed drugs for FOP seem encouraging, thorough clinical trials are essential [111,146]. Modern tools like computational biology and cell models from patients can help to speed up this process. Collaborations between researchers, patient groups, and regulators can further streamline drug approval. Repurposing drugs presents an efficient and promising path for better understanding and treating FOP. 

## 7. Impediments and Innovations for Clinical Trials for FOP

The structural and epistemological complexities in executing clinical trials for FOP, or any ultra-rare disease, are enormous [39,147]. These obstacles include a dearth of standardized natural history data to inform trial designs, the scarcity of validated and surrogate outcome measures, and diminutive patient populations, rendering traditional large-scale randomized control trials (RCTs) infeasible [39,147,148]. RCTs serve as the acme of empirical efficacy assessment in clinical research, primarily due to their capacity for minimizing selection bias and distributing potential confounders evenly across study groups. Nevertheless, the paucity of large cohorts in ultra-rare conditions like FOP disrupts the statistical robustness of RCTs. When samples are small, the stochastic noise associated with inter-individual differences amplifies, effectively attenuating the trial’s statistical power and, therefore, increasing the risk of Type II errors. 

Conversely, uncontrolled trials, which juxtapose interventional outcomes against the known natural history of a disease, offer a somewhat more practical design for conditions like FOP. However, this utility is constrained by the still-evolving understanding of FOP’s natural history and its marked inter-individual heterogeneity. Furthermore, subjects in natural history studies may under-report adverse events, engendering a bias that can discredit the risk–benefit profile of a new therapeutic agent.

To address these challenges, future FOP trials should incorporate adaptive designs and Bayesian statistical methods to rectify these pitfalls [149,150]. Adaptive designs are particularly valuable in early-phase clinical trials, where they allow modifications to trial procedures based on interim results [149,150,151]. This maximizes the utility of data from small sample sizes while maintaining the trial’s integrity and is particularly useful in early-phase clinical trials [151]. For instance, a recent study re-designed the High-Frequency Oscillation in Acute Respiratory distress syndrome (OSCAR) trial using Bayesian adaptive design methods to allow the possibility of early stopping for success or futility [149]. The study constructed several alternative designs and studied their operating characteristics via simulation. The virtual re-executions showed that the Bayesian sequential approach and original OSCAR trial yielded similar trial conclusions. However, using a Bayesian sequential design could have led to a reduced sample size and earlier completion of trial 1 [151]. Bayesian statistics takes advantage of prior data to inform current analysis, enhancing the interpretative power of trials with smaller cohorts. Bayesian methods can also be used to estimate design operating characteristics of Bayesian adaptive trials [152,153]. For instance, a recent study proposed an approach to design adaptive clinical trials without needing to specify the complete data-generating process. To facilitate this, they considered a general Bayesian framework where inference of the treatment effect on a time-to-event outcome could be performed via the partial likelihood [154]. 

Leveraging innovative trial designs, e.g., master protocols and complex adaptive designs in conjunction with a Bayesian approach, may help to reduce sample size, select the correct treatment and population, and accurately and reliably assess the treatment effect in rare disease settings like FOP. 

A multitude of drugs targeting Activin A and BMP signaling pathways are currently in various phases of clinical trials, ranging from Phase 1 to Phase 3 [Table 2]. Each trial, while conforming to a randomized, double-blind, and placebo-controlled design, features unique arm configuration and primary endpoint measures, primarily focused on the volume of new HO lesions. However, these trials are not without limitations. Issues, e.g., patient recruitment, disease heterogeneity, a lack of reliable biomarkers, and ethical concerns regarding placebo controls, constitute formidable challenges.

Of note are the ethical implications surrounding the use of placebo groups in a disease as debilitating as FOP. The ethical aspects become particularly challenging when one considers the lifelong morbidity associated with each ossification event, thus raising the question of whether a placebo-controlled design can ever be ethically justifiable in this context [148]. The daunting obstacles inherent to FOP clinical trials epitomize the methodological and ethical complexities plaguing ultra-rare disease research. While no panacea exists for surmounting these challenges in their entirety, innovative trial designs, sophisticated statistical methods, and a conscientious approach to ethical considerations can ameliorate these issues. FOP trials serve as a crucible, testing the adaptability and ingenuity of clinical research methods for ultra-rare diseases [34,147]. They force a reconceptualization of traditional paradigms, mandating a fusion of scientific rigor and ethical sensibility, all while maintaining an unwavering focus on the exigencies of patient welfare.

## 8. Conclusions

In summary, our manuscript offers a comprehensive investigation into the evolving landscape of FOP research and therapy. We embark on a journey through the traditional therapeutic models and pharmacological interventions and culminate with the advent of immunotherapy as a potential game-changer for FOP treatment. As we show, the disease’s complex pathophysiology poses both challenges and opportunities for targeted therapies, with immunotherapy emerging as a particularly promising approach. However, while the scientific frontier appears expansive, our exploration of clinical trial methodologies elucidates numerous hurdles that need overcoming—these range from statistical challenges posed by small sample sizes to the ethical implications of placebo-controlled trials in a disease as debilitating as FOP.

Our manuscript serves not just as a summary of current research but as an urgent call to action. It underscores the need for methodological innovation in trial design and the adoption of advanced statistical approaches that can accommodate the unique constraints of ultra-rare disease research. By intertwining rigorous scientific scrutiny with ethical considerations, we can hopefully move closer to identifying therapies that not only alleviate symptoms but genuinely improve the lives of FOP patients.

It is imperative to maintain an unwavering commitment to ethical clinical research practices, balancing both the exigencies of patient welfare and scientific rigor. In the endeavor to transform the lives of individuals affected by FOP, this manuscript aims to catalyze a concerted effort among clinicians, researchers, and bioethicists to navigate the challenges ahead. The road to efficacious FOP treatment is steep and fraught with obstacles, but it is a path that tests the resilience, innovation, and adaptability of the scientific community at large. We conclude that FOP serves as both a crucible and a beacon, guiding the evolution of research methodologies and ethical frameworks for ultra-rare diseases. The pursuit of a cure for FOP, thus, holds the potential to revolutionize not only the field of rare disease research but also the broader landscape of medical science.

## Figures and Tables

**Table 1:** Overview of genetic approaches for treating FOP. Gene editing focuses on directly correcting the ACVR1/ALK2 gene mutations at the DNA level using gene editing tools like CRISPR-Cas9, while gene addition aims to introduce healthy copies of the *ACVR1* gene into the cells. Gene silencing explores either the full inactivation of the *ACVR1* gene or selective suppression of only the mutated allele. The gene replacement approach integrates gene addition and gene silencing techniques for a multi-faceted therapeutic solution.

Therapeutic Strategy	Objective	Molecular Target	Anticipated Outcome
Gene editing	Rectification of mutations in the *ACVR1* Gene using strategies like CRISPR-Cas9	DNA	Exclusive expression of the corrected ACVR1/ALK2 protein
Gene addition	Introduction of healthy, functional *ACVR1* gene copies	DNA, mRNA	Competition between newly added functional ACVR1/ALK2 and existing mutant forms
Gene silencing	Full inactivation or allele-specific suppression of *ACVR1*	mRNA	Full inactivation may lead to unintended physiological ramifications; allele-specific suppression selectively diminishes the expression of the mutant *ACVR1* gene
Gene replacement	Synchronizing gene addition and gene silencing	mRNA	Allele-specific suppression reduces mutant *ACVR1* expression, while the addition of functional *ACVR1* compensates for the deficiency in functional ACVR1/ALK2

**Table 2:** Ongoing interventional clinical trials for FOP. A brief summary of ongoing interventional clinical trials focused on therapeutic interventions for FOP (as of 18 September 2023).

ClinicalTrials.gov Identifier	Study Title/ Sponsoring Entity	Intervention	Participants	Primary Outcome
NCT05394116	OPTIMA: a study to assess the safety, tolerability, and efficacy of garetosmab versus placebo administered intravenously (IV) in adult participants with fibrodysplasia ossificans progressiva (FOP) by Regeneron Pharmaceuticals	Garetosmab (REGN2477)	Adults, both sexes	Quantification of newly developed HO lesions via adjudicated CT scans; the occurrence and gradation of special-interest treatment-emergent adverse events (AESIs)
NCT05039515	FALKON: study to assess the effectiveness and safety of two dosage regimens of oral fidrisertib (IPN60130) for the treatment of fibrodysplasia ossificans progressiva (FOP)/ Clementia Pharmaceuticals Inc.	Fidrisertib (IPN60130)	Ages 5 and above, both sexes	Yearly alteration in HO volume, measured through low-dose WBCT (head excluded); incidence of adverse events/serious adverse events (AEs/SAEs); baseline deviation in critical laboratory parameters (hematology, biochemistry, urinalysis); baseline changes in physical examinations; alterations in vital signs and ECG readings from baseline
NCT04307953	STOPFOP: saracatinib trial to prevent FOP by Amsterdam UMC	Saracatinib (AZD0530)	Ages 18 to 65, both sexes	Objective variance in heterotopic bone volume, assessed via low-dose whole-body CT, across both study arms during the initial 6-month RCT period
NCT05090891	PROGRESS: To assess the efficacy, safety, and tolerability of INCB000928 in participants with fibrodysplasia ossificans progressive by Incyte Corporation	Zilurgisertib (INCB000928)	Ages 12 to 99, both sexes	Comprehensive assessment of new HO volume
NCT05027802	PIVOINE: a rollover study to further evaluate the safety and efficacy of palovarotene capsules in male and female participants aged ≥14 years old with fibrodysplasia ossificans progressiva (FOP) who have completed the relevant parent studies by Ipsen Biopharmaceuticles	Palovarotene	Ages 14 and above, both sexes	Incidence and categorical elucidation of all serious and non-serious treatment-emergent adverse events (TEAEs), irrespective of their causal relation to the study intervention

## Data Availability

Not applicable.
